# The Role of Physicians in Expanded Access to Investigational Drugs: A Mixed-Methods Study of Physicians’ Views and Experiences in The Netherlands

**DOI:** 10.1007/s11673-021-10090-7

**Published:** 2021-02-15

**Authors:** Eline M. Bunnik, Nikkie Aarts

**Affiliations:** Department of Medical Ethics, Philosophy and History of Medicine, Erasmus MC, University Medical Centre Rotterdam, Wytemaweg 80, 3015 CN Rotterdam, The Netherlands

**Keywords:** Expanded access, Compassionate use, Physicians’ attitudes, Clinical decision-making, Mixed methods, Ethical issues, Moral responsibilities

## Abstract

Treating physicians have key roles to play in expanded access to investigational drugs, by identifying investigational treatment options, assessing the balance of risks and potential benefits, informing their patients, and applying to the regulatory authorities. This study is the first to explore physicians’ experiences and moral views, with the aim of understanding the conditions under which doctors decide to pursue expanded access for their patients and the obstacles and facilitators they encounter in the Netherlands. In this mixed-methods study, semi-structured interviews (n = 14) and a questionnaire (n = 90) were conducted with medical specialists across the country and analysed thematically. Typically, our respondents pursue expanded access in “back against the wall” situations and broadly support its classic requirements. They indicate practical hurdles related to reimbursement, the amount of time and effort required for the application, and unfamiliarity with the regulatory process. Some physicians are morally opposed to expanded access, with an appeal to safety risks, lack of evidence, and “false hope.” Some of these moral concerns and practical obstacles may be essential targets for change, if expanded access to unapproved drugs is to become available for wider groups of patients for whom standard treatment options are not—or no longer—available, on a more consistent and equal basis.

## Introduction

Doctors may, under strict conditions, prescribe investigational drugs that are not (yet) approved for marketing to patients who run out of standard treatment options. This is generally referred to as “expanded access.” Although terminologies and regulatory routes differ (Kimberly et al. 2017), in many countries, expanded access is allowed through programmes either for (large) groups of patients or for individual patients (or small groups of patients). In the Netherlands, these programmes are referred to as “compassionate use” and “named-patient” programmes, respectively (table 1). The criteria for expanded access are similar in most countries: patients qualify when they a) are suffering from a serious and/or life-threatening disease, b) have exhausted standard treatment options, and c) cannot enrol in a clinical trial (e.g., because there are no clinical trials in their geographical area or because they fail to meet the inclusion criteria). The treating physician must decide based on limited data whether the investigational drug might offer a reasonable chance at medical benefit which outweighs the risk. In addition, in a few countries, including the United States, Spain, and Italy, approval from an institutional review board (IRB) is required (Borysowski et al. 2017). IRB approval is not required in the Netherlands.

**Table 1:** Expanded access in the Netherlands

**Compassionate use programme**	**Named-patient programme**
Initiated by the pharmaceutical company For (large) group of patients Usually after successful completion of phase III clinical trial Approval by Medicines Evaluation Board	Initiated by the treating physician For individual patient or small group of patients Usually possible after phase I/IIa clinical trials Approval by Health Inspectorate

On May 30, 2018, President Trump signed a federal Right-to-Try bill into law, which allows pharmaceutical companies under specific conditions to provide patients with unapproved drugs without having to apply for authorization by the Food and Drug Administration (FDA). In the United States of America, the use of unapproved drugs is not common. The FDA handles around a thousand expanded access investigational new drug (IND) applications per year (Jarow et al. 2016)—which may, however, cover multiple patients per IND—for non-biological drugs only. Although the American Right-to-Try campaign aims at making unapproved drugs available to wider groups of patients with unmet medical needs, the bills have been heavily criticized (Bateman-House and Robertson 2018; Holbein et al. 2015). Foremost, it is not the FDA that keeps patients in dire circumstances from accessing unapproved drugs; over the course of ten years, the FDA approved 99.3 per cent of the almost nine thousand requests it received (Jarow et al. 2016). Rather, pharmaceutical companies may be unwilling or unable to supply drugs outside of clinical trial settings (Darrow et al. 2015; Falit and Gross 2008), often at no cost (Miller et al. 2017). Right-to-Try legislation does little to change this, as it does not force or stimulate pharmaceutical companies to supply investigational drugs, nor does it provide mechanisms, for instance, for outcome data collection, which might render it more attractive for companies to do so. Also, many physicians—and patients—are unfamiliar with expanded access and may not know that it exists or how to pursue it (Bateman-House and Robertson 2018).

Treating physicians have key roles to play in expanded access, by identifying promising new drugs, informing patients about opportunities for trying these new drugs, and preparing the application. Confronted with challenges ranging from reimbursement and liability issues to lack of data on the safety and efficacy of the new drug and the uncertainty whether the pharmaceutical company will release the drug, expanded access “can feel like navigating uncharted waters” (Bateman-House 2016, ¶1). To doctors, expanded access may be “a complex, labyrinthine process” (Jerome et al. 2016, 305) and involves an extensive administrative burden: the communication and negotiation with the pharmaceutical company, the writing of a medical case report indicating the rationale for trying expanded access as part of the application process to the regulatory authorities, the obtaining of informed consent by the patient, the reporting of adverse events and possibly outcome data (Darrow et al. 2015). A few years ago, the FDA made changes to facilitate its procedures for applying for access (Gaffney 2015). However, a recent survey study suggests that for paediatric oncologists in the United States, the—actual or perceived—significant burden of administrative responsibility may still constitute a barrier to the pursuit of compassionate use (Moerdler et al. 2019).

Very little empirical research has been conducted into physicians’ views and experiences with regard to expanded access to investigational drugs. This mixed-methods study is the first to explore physicians’ experiences with expanded access and their reasons for pursuing it (or not) for their patients through a series of individual interviews and a questionnaire among medical specialists across the Netherlands. The Netherlands has a system of universal health coverage and mandatory health insurance. Health insurers must reimburse approved treatments and generally do not cover the costs of expanded access. This may explain in part why the uptake of expanded access in the Netherlands is similarly low as in the United States; it is estimated to be between one hundred and two hundred applications per year (de Visser 2016). In countries such as France and Turkey, where investigational drugs are routinely reimbursed through the national healthcare system, they are more frequently prescribed (Degrassat-Théas et al. 2013; Vural et al. 2012). In France, for instance, over 21,000 patients gain access to investigational drugs through its Autorisation Temporaire d’Utilisation (ATU) programme every year (Agence Nationale de Sécurité du Médicament et des produits de santé [ANSM] 2018). The results of this study will be relevant foremost to ethical and policy discussions on expanded access in the Netherlands but may also be of interest to those working in countries such as the United States and many other European countries, in which regulatory routes for expanded access in some respects are similar.

## Methods

A mixed research design was used: individual interviews were conducted with medical specialists across the Netherlands to explore their views and experiences regarding the use of unapproved investigational drugs, followed by a questionnaire among medical specialists to reach a broader audience and to substantiate the results from the interviews. We received a waiver from the research ethics review committee of Erasmus MC (MEC-2016-275), as the research does not fall within the scope of the Dutch Medical Research Involving Human Subjects Research Act. All interview respondents provided oral informed consent for recording and publication. The study was conducted in accordance with the COREQ guidelines (Tong et al. 2007). A COREQ checklist can be found in the Appendix.

### Selection of Participants

Medical specialists were approached by email with an invitation for an individual interview. Specialists were selected who would likely care for patients for whom standard treatment options may not (or no longer) be available—in the fields of neurology, psychiatry, endocrinology, and (sub-specializations of) oncology—through purposive sampling to secure a wide range of opinions. Physicians were recruited through hospital websites—if their email addresses were publicly available—or scholarly publications or were contacted through the researchers’ networks or snowball sampling. Specialists worked in various types of hospitals (i.e., rural hospitals, teaching hospitals, and academic hospitals) in different regions of the Netherlands. There are three types of hospitals in the Netherlands: rural hospitals that offer standard care for less complex health problems (“second line” care), teaching hospitals that are larger and affiliated with academic hospitals, which train healthcare professionals and offer both standard and specialized (“third line”) medical care, and academic hospitals that are connected to major universities and offer both standard and highly specialist referral medical care.

After completion of the interviews, we developed a quantitative online questionnaire to supplement the qualitative interview data. We cast a wide net in the attempt to reach physicians who had experience with expanded access. IMS Health, a health market intelligence agency (which has since merged into IQVIA, a contract research organization (IQVIA 2020)) provided us with email addresses of a large number of Dutch medical specialists registered under selected specializations—oncology, endocrinology, gastroenterology, haematology, internal medicine, neurology, neurosurgery, infectious disease medicine, and respiratory medicine. Personal invitations were distributed among 5724 physicians and physicians-in-training between October 2016 and January 2017 through email and/or postal mail. One reminder was sent by email three weeks after the initial invitation. In February 2017, one month after the latest mailing, the questionnaire was made publicly accessible online and brought to the attention of physicians in the researchers’ networks and through Twitter.

### Interviews

Interviews were conducted with the help of a semi-structured interview guide and included the following topics: characteristics of the patient population and their standard treatment options; non-standard options; views and decision-making with regard to clinical trials, off-label use of drugs, compassionate use or named-patient programmes; communication and shared-decision making; moral responsibilities of the physician; and demographic characteristics. After the first two interviews, minor adjustments were made to the order of the questions in the interview guide to improve the course of the interview, allowing it to converge more slowly towards the “last-ditch option” of expanded access. The individual interviews took place at the interviewees’ workplace or home, lasted on average between forty-five and ninety minutes, and were conducted by NA and/or EB between December 2015 and May 2016. An English translation of the interview guide can be found in the Appendix.

### Questionnaire

The questionnaire was developed in LimeSurvey and included similar topics. Participants were asked to answer mostly multiple-choice questions and some free-text questions and to rate six statements about investigational drugs and physicians’ responsibilities. Free-text questions were asked if more detailed information was needed, for example about physicians’ experiences with investigational drugs, or if respondents chose “other” in response to multiple-choice questions. Completing the questionnaire took around ten minutes. A pilot version was tested by seven medical specialists with different backgrounds and two qualitative research experts and adjusted accordingly. An English translation of the questionnaire can be found in the Appendix.

### Analysis

All interviews were audiotaped and transcribed verbatim. Coding and analysis were performed using NVivo version 11. Interviews were coded and analysed using a constant comparative method, in which codes were assigned and (re)grouped into categories (themes) through multiple readings of the interviews (Glaser and Strauss 1999; Bradley et al. 2007). A code tree with multiple levels of codes was developed during independent analyses of the first three interviews by NA and EB. Discrepancies were discussed until consensus was reached. Remaining interviews were coded by NA. After the analysis of the twelfth interview, theoretical saturation was reached, and the code tree was no longer adapted. Quotes from the interviews were translated from Dutch by EB.

The questionnaire resulted mainly in quantitative data. Descriptive statistics were applied to analyse the data using SPSS, IBM Statistics, version 23.0. Valid percentages (i.e., percentages after missing data were excluded) are presented in tables for responses to multiple choice questions. Free-text responses were categorized into themes and used for illustration.

## Results

### Characteristics of the Interviewees

In total, fourteen interviews with medical specialists were conducted. Interviewees were specialists in oncology (n = 7) with sub-specializations in hematologic, gynaecologic, gastrointestinal, and lung cancers, neurology (n = 3), endocrinology (n = 2), and psychiatry (n = 2). Nine worked in academic hospitals, five worked in rural or teaching hospitals. Six out of fourteen interviewees were female, and the median age was 50.0 years (interquartile range 41.8–55.3). All but one physician were (sometimes) involved in clinical research, participating in multicentre trials conducting observational studies or early-phase clinical trials. The interviewees treated patients for whom curative or satisfactory treatment options were lacking; their patients were either without any standard treatment options altogether or without any standard treatment options that sufficed. Interviewees’ characteristics are reported in Table 2.

**Table 2:** Characteristics of the interviewees and respondents

	**Study population interviewees** **N = 14** **n (%)**	**Study population respondents** **N = 90** **n (%)**
**Female**	6 (42.9)	31 (36.5)
**Male**	8 (57.1)	54 (60.0)
**Age**		
< 30 years	0 (0.0)	2 (2.3)
31 – 40 years	1 (7.1)	33 (38.4)
41 – 50 years	6 (42.9)	17 (19.8)
51 – 60 years	7 (50.0)	24 (27.9)
> 60 years	0 (0.0)	10 (11.6)
**Type of employment**		
Medical specialist in peripheral hospital	0 (0.0)	23 (25.6)
Medical specialist in leading clinical hospital	5 (35.7)	23 (25.6)
Medical specialist in academic hospital	9 (64.3)	39 (43.3)
Medical specialist in training	0 (0.0)	5 (5.6)
**Specialization**		
Internal medicine, oncology	7 (50.0)	14 (15.6)
Internal medicine, other specializations ^a^	2 (14.3)	33 (36.7)
General internal medicine	0 (0.0)	16 (17.8)
Neurology	3 (21.4)	18 (20.0)
Other ^b, c^	2 (14.3)	9 (10.0)

### Characteristics of the Questionnaire Respondents

The response rate for the questionnaire following our mailing efforts was extremely low: the questionnaire was sent to 5724 physicians and physicians-in-training, and we received sixty-one complete responses after the first round of email and postal mail. After we sent reminders and made the questionnaire publicly accessible, another sixty-eight respondents completed the questionnaire. As the characteristics of the respondents in the first and second round did not differ, the data were grouped and analysed together. In total, 129 respondents completed the questionnaire. Thirty-nine respondents were excluded as they did not work in a hospital (n = 10) or did not lack standard treatment options that could cure their patients or prolong their lives. A total of ninety respondents matched our target population of medical specialists in rural, teaching, or academic hospitals who would be in a position to consider expanded access. Respondents’ characteristics are reported in Table 2.

### Themes

We identified five themes: limited knowledge of and experience with expanded access, disparate attitudes towards expanded access, reasons for expanded access, reasons against expanded access (including practical hurdles), and views on physicians’ responsibilities regarding expanded access.

#### Limited Knowledge of and Experience with Expanded Access

Apart from the psychiatrists and the gynaecologist, most interviewees had had some experience with compassionate use. Compassionate use programmes had mostly been run post-trial, to bridge the gap between completion of the clinical trial and market authorization (see Table 1). When interviewees had applied for compassionate use, they had treated a handful of patients, at most. The familiarity with named-patient programmes was much more limited. Some interviewees (n = 3) had never heard of this route to access an investigational drug:

Well, barely. Uhm … I never understand completely, what it is about. (neurologist)

No, I only remember that I had to sign a statement … about the unapproved drug so that the pharmacy could import it. It was very complicated. I had to write down a motivation for why I did that, and that I would accept the responsibility for the administration and the side effects of that drug. I remember that it was one piece of paper … This was fifteen years ago. I have not done it since. (endocrinologist)

Other interviewees did have some experience requesting a drug through named-patient programmes. Their total experience typically consisted of one or a few individual requests. Physicians working in academic hospitals mentioned specific genetic mutations in tumour biology and exclusion from a clinical trial as reasons for having pursued named-patient expanded access for their patients in the past.

According to our interviewees, only a small percentage of patients ask for expanded access. These patients may have heard about investigational treatment options in the news, on the Internet, or from patient advocacy groups. The largest group of patients, however, is worn out by the time they reach the end of a standard treatment trajectory and no longer willing to try. A doctor can usually tell who among his or her patients will continue to look for other options. These are commonly the patients who are proactive and habituated to being in charge:

You know, beforehand you can tell which ones are the fighters, right, the ones who will never stop. You know that, they will go down fighting, you know it. (oncologist)

#### Experiences with the Named-Patient Programme: Questionnaire Results

In our questionnaire, nineteen respondents (21.1 per cent) reported having had experience with requesting an investigational drug through a named-patient programme. All but one respondent had been able to acquire the drug for their patient, and all but two claimed that patients had benefited in some way (mostly medically) from use of the investigational drug. The costs for the investigational drug were covered by the pharmaceutical company (n = 6), hospital (n = 4), health insurer (n = 7), or by the patient (n = 1). Respondents’ experiences with the application process were variable: seven respondents experienced the process as extensive and cumbersome, but seven other respondents said it was quick and easy. One respondent had varying experiences. In free-text responses, a gastro-intestinal oncologist and an internal oncologist commented, respectively: “[It took] paperwork … But in the end, it did happen,” and “[It was] a lot of administrative hassle, therefore took a long time.” Two respondents from academic hospitals specifically mentioned that they were supported by the hospital pharmacist in applying for the unapproved drug. Their experience of the process was positive. One respondent noted: “Good. The pharmacist at our department arranges these things.”

#### Disparate Attitudes Towards Expanded Access

Not only was the level of knowledge and experience with expanded access highly variable across interviewees, so too were their views on the desirability of expanded access. They were more willing to consider applying for existing compassionate use programmes run by pharmaceutical companies than to consider requesting expanded access for individual patients on their own initiative. There were interviewees who were categorically opposed to expanded access, citing the principles of evidence-based medicine. When pressed, they explained that they made clinical decisions in accordance with clinical guidelines and would only prescribe drugs that were sufficiently tested, approved for marketing, and suggested in clinical guidelines issued by professional organizations.

Others were sceptical that new drugs would bring substantial benefits to patients:

I have never felt I was held back, I have not at all had the impression that there was a pot of gold at the rainbow’s end, and I couldn’t reach it. Not at all. (oncologist)

On the other hand, four interviewees were adamant proponents of expanded access—under strict conditions—and felt it was part of the core of their care provision for patients. They considered it their task to seek beyond standard treatment options or clinical guidelines when standard options had run out but believed that not all physicians would do the same.

It is doctor-dependent and context-dependent and knowledge-dependent and however much we would want to have it otherwise, it is true that these out-of-standard procedures, well, that [access to these procedures is] not levelled. No, not all, not everyone is equal. Not everyone has equal chances. It is what it is. (oncologist)

#### Disparate Attitudes Towards Expanded Access: Questionnaire Results

**Fig. 1:**
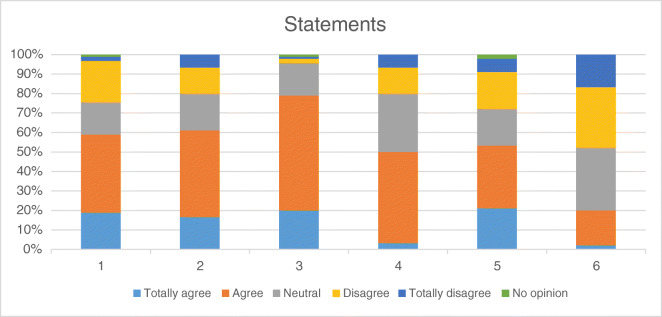
To what extent do respondents agree with statements on moral responsibilities?

In our questionnaire, fifty-three respondents (58.9 per cent) either agreed or strongly agreed with the statement that physicians should *not* prescribe investigational drugs outside of clinical trials (Fig. 1).

Statements:

1. I believe that physicians should not prescribe investigational drugs outside of clinical trials.
2. I believe that it is the responsibility of a physician to refer patients without curative or life-prolonging treatment options to (highly) specialized care.
3. I believe that it is the responsibility of a physician to identify clinical trials in which a patient without curative or life-prolonging treatment options may possibly be enrolled.
4. I believe that physicians should inform their patients about the options for accessing investigational drugs.
5. I believe that the decision to use an investigational drug should be made by the patient him- or herself.
6. I believe that it is the responsibility of a physician to look, at his/her own initiative, for investigational treatments for patients without curative or life-prolonging treatment options.

#### Reasons for Expanded Access

Interviewees mentioned multiple reasons why they would prescribe investigational drugs. The most important reason was that it might help their patient—for example, by improving their prognosis or quality of life. Interviewees indicated that they would need scientific evidence to determine the balance of potential risks and benefits. Respondents had different standards for what is “enough information”: some required “positive phase 3 trials” (oncologist 1), whereas others would consider an investigational drug based on “a sound biological rationale” (oncologist 2) or “clinical experience after some time without head-to-head trials” (neurologist 1)

[I should be] convinced of the possible utility [of the drug] for this patient. Maybe in the absence of alternatives with a prognosis that does not look very favourable ... Well, in the back-against-the-wall situation, I should be at ease with the little information that I’ve got on the potential side effects. (endocrinologist)

Interviewees often spoke of “being with their backs against the wall” as a reason to consider expanded access: they might pursue it (only) if there were no other options for a patient. Two interviewees, however, mentioned that under very exceptional circumstances, they would prefer to prescribe an investigational drug *before* standard of care, thereby diverging from one of the classic conditions for expanded access (i.e., that there should be no approved alternatives). If, for instance, standard of care consisted of combination chemotherapy, which would wear down patients and offer a poor prognosis, then it might be better to start with the investigational drug straightaway *instead of* trying standard of care first. Of course, these interviewees thought it preferable to enrol a patient in a clinical trial, but if trials were non-existent, they would wish to resort to applying for a named-patient programme before trying—ineffective and harmful—standard of care.

In general, physical fitness was an important condition for interviewees: patients would have to be in a relatively good condition to try an investigational drug. At the end of a standard treatment trajectory, patients often have long medical histories behind them and are not physically fit enough to try investigational drugs.

Sometimes you know that you’ve got four lines of treatment, then, then you know, like, well, if I go and try all these lines of treatment first, the patient will become cooked-through like butter. Then he will not be able to try the investigational treatment. So then I [would want to] refer him sooner. (oncologist)

Interviewees needed to feel comfortable when prescribing an investigational drug. For some, this required not only a sufficient level of evidence about the risks and benefits but also the favourable opinion of their peers. Interviewees would prescribe investigational drugs much more readily when the medical profession made positive statements about the drug or when they felt supported by colleagues. It is considered important to discuss expanded access with colleagues from in- or outside the hospital, beforehand. One interviewee reported that after discussing with colleagues,

… now I had the feeling, I had enough external information and I had, well, I had enough safeguards. So I did not have the idea that … I was doing something like that [expanded access] on my own, without anyone looking in. (psychiatrist)

In the process of expanded access, the help of a hospital-based pharmacist was considered very valuable. Some interviewees mentioned that it was the hospital-based pharmacist who had actually brought up the possibility of expanded access and guided them through the application process.

#### Reasons for Expanded Access: Questionnaire Results

Questionnaire respondents were also asked whether and under what conditions they would consider prescribing an investigational drug and what their reasons were for prescribing investigational drugs outside of clinical studies. A slight majority of forty-seven respondents (52.2 per cent) would consider prescribing investigational drugs through compassionate use or named-patient programmes or would support referral to a foreign country (based on question 13 of the questionnaire, see Appendix). Their most important reasons for pursuing expanded access pertained to patients’ health: to improve their health, to prolong their lives, or to improve quality of life (Table 3). Also, confidence in the effectiveness of the investigational drug was an important condition and the fact that a pharmaceutical company would offer a compassionate use programme. None of the respondents would pursue expanded access to provide hope, to protect the physician–patient relationship, or “because the patient will pay for it him- or herself.”

**Table 3:** Reasons for physicians to prescribe an investigational drug outside a clinical study

**Reasons**	**Study population** N=43* n (%)
To improve quality of life	24 (55.9)
Gain in health	22 (51.2)
Possible prolongation of life	19 (44.2)
Confidence in the effectiveness	18 (41.9)
Pharmaceutical company offers compassionate use programme	14 (32.6)
Request from the patient	8 (18.6)
‘With my back against the wall’	7 (16.3)
Collection of observational data	4 (9.3)
There are none	3 (7.0)
Request from the family of the patient	1 (2.3)
To give hope	0 (0.0)
To protect the physician-patient relationship	0 (0.0)
Patients pays for the drug himself	0 (0.0)
Other (“for rare condition with clear pathophysiological basis”)	1 (4.7)

*Reasons for prescribing investigational drugs were mentioned by 43 of 47 respondents who would consider prescribing an investigational drug. Some respondents mentioned multiple considerations.

#### Reasons Against Expanded Access

Interviewees mentioned a wider range of reasons not to prescribe an investigational drug. Lack of scientific evidence was most important in physicians’ decisions against expanded access. Interviewees who were hesitant about expanded access were sceptical about the limited data on effectiveness and safety that is commonly available after phase II clinical trials, commenting that many drugs fail in later stages of drug development. These interviewees required more certainty regarding the drug’s potential benefit before they would consider exposing their patients:

After all, the one receptor is not the other. And the fact that something works in A does not mean that it works in B. And that a phase I study has been conducted somewhere, does not mean anything. At that point you are close to being nowhere. (oncologist)

Moreover, according to interviewees in various medical fields, there are very few (or no) break-through life-saving drugs in the pharmaceutical pipeline. Interviewees believed that most new drugs will not make much of a difference to patients and that the possible medical benefit will likely be marginal. Some interviewees believed that drugs that are exceptionally beneficial and/or life-saving will be evaluated fast-track and will be quickly approved for marketing through conventional channels and made available to patients. Expanded access would thus not be necessary for patients:

The gains of these drugs are of course limited. If they were very spectacular, I would have known about them. That happened once with Herceptin, in breast cancer. This is the only example that I can remember: the gains were so high that everyone thought it was unethical to wait until the authorization process had been completed. But even then, you could question [whether expanded access is appropriate]. (oncologist)

By not prescribing or proposing investigational drugs, interviewees say they are protecting their patients against “false hope.” Every subsequent treatment option brings new hope for a patient. When that hope is based on only limited data, showing limited potential for medical benefit, it is considered false. Additionally, drugs are never without risks. The principle of *primum non nocere* entails that patients should be protected from such risks. A respiratory physician states that, often, patients think that

… it does not hurt to try. But that is of course not true, that is simply not true … Of course, you derive hope from [investigational drugs]. And as a doctor, too, to be honest, I always hope. (respiratory physician)

Patients may be exposed to false hope, burdens, and possible risks when trying investigational drugs.

Interviewees emphasized that it would be unethical to expose patients to such risks without learning from it. Data collection was considered an important condition for expanded access, also in light of the resources (e.g., physicians’ time and effort, the expense of the compound) dedicated to a single patient. Future patient populations should also benefit from the knowledge that is gathered from individual cases of expanded access:

That I find may be the most important thing: that if we do things outside of clinical trials, that we register accurately what we do, so that we can learn from it. (oncologist)

#### Practical Hurdles

What kept interviewees from pursuing expanded access for their patients with unmet medical needs were most often *reasons* but sometimes also practical *obstacles*. Interviewees mentioned lack of experience, the administrative burden, the time and effort that it takes to request expanded access, problems with funding or reimbursement, and lack of cooperation by other parties, notably pharmaceutical companies or hospital management. An oncologist states: “No, with all of those formal boxes you need to tick and God knows with all the bureaucratic horror. No, there are a lot of bells and whistles attached to it.” A respiratory physician states:

The tempo and the time and energy that you have to put into [expanded access]. That is the biggest problem, right? Because ultimately, you prefer spending most of your time by the patient’s bedside. (respiratory physician)

Funding issues are frequently mentioned as a major obstacle. An interviewee explains:

Then it goes to the departmental budget. And you cannot turn to the health insurer either. You can try, but in our experience, you immediately get: zero. They simply look at: is this an approved drug? If it is not approved, it does not fall in the basic package, we do not need to reimburse it. (endocrinologist)

#### Reasons Against Expanded Access: Questionnaire Results

All respondents, including respondents who had never considered expanded access, were asked about the most important reasons *not* to seek expanded access. For questionnaire respondents, the most frequently selected reasons were safety risks, “false hope,” the low chance of effectiveness, the “experimental nature” of the drug, and the fact that there is no systematic data collection (Table 4).

**Table 4:** Reasons for physicians not to prescribe an investigational drug outside a clinical study

**Reasons**	**Study population** N=90* n (%)
Safety risks	37 (41.1)
False hope	35 (38.9)
Low chance at effectiveness	31 (34.4)
Experimental nature of the drug	30 (33.3)
No systematic data collection	23 (25.6)
Drug is not recommended in clinical guidelines	20 (22.2)
No reimbursement of the drug	20 (22.2)
There are no promising new drugs	19 (21.1)
Costs of the drug	19 (21.1)
I wait until a clinical trial opens up	18 (20.0)
Unfamiliar with the procedure	16 (17.8)
Possible legal consequences	14 (15.6)
A lot of work	9 (10.0)
I did not know it was possible	8 (8.9)
Not enough time	7 (7.8)
Health inspectorate will not approve	4 (4.4)
There are none	2 (2.2)
Other**	6 (6.7)

*Some respondents mentioned multiple considerations.

**Mostly respondents who refer patients on or feel that the situation is not applicable to them.

Moreover, some of the reasons selected were actually practical obstacles. Respondents were unfamiliar with the procedure (17.8 per cent), did not know it was possible (8.9 per cent), indicated that it was a lot of work (10.0 per cent) and that they did not have enough time to initiate an application process (7.8 per cent) (Table 4).

The subgroup of respondents who would consider or had first-hand experience with prescribing or referring for an investigational drug indicated that they would expect or had experienced obstacles mostly related to reimbursement. Almost half of the respondents (46.5 per cent) indicated that the health insurance agency would not reimburse the drug, while a quarter indicated that the hospital would not (Table 5). Another major hurdle for respondents was the time it takes to apply for expanded access: 39.5 per cent of respondents indicated that the application takes too much time. Other obstacles included policies at the hospital that discouraged doctors from using unapproved drugs, refusals by pharmaceutical companies, and lack of familiarity with the regulation (Table 5).

**Table 5:** Obstacles experienced by physicians when prescribing investigational drugs

**Obstacles**	**Study population** N=43*
Insurer does not reimburse the drug	20 (46.5%)
Application takes too much time	17 (39.5%)
Unfamiliarity with the regulation	11 (25.6%)
Hospital does not reimburse the drug	10 (23.3%)
Hospital discourages the use of investigational drugs	8 (18.6%)
Pharmaceutical company does not provide the drug	7 (16.3%)
Pharmaceutical company charges too much money	6 (14.0%)
No new promising drugs	5 (11.6%)
Health inspectorate does not provide a license or too late	5 (11.6%)
Other**	5 (11.6%)
No obstacles	2 (4.7%)

*Study population are respondents who replied that they consider prescribing investigational drugs outside of clinical trial settings. Some respondents mentioned multiple obstacles.

**Other includes: only in clinical trials or negative views of expanded access.

#### Views on Physicians’ Responsibilities Regarding Expanded Access

When confronted with the question: “What do you do when standard treatment options have been exhausted? What do you tell your patient?” interviewees often considered their moral responsibilities. Interviewees in rural hospitals felt that when they run out of standard treatment options, they must refer patients to experts in academic hospitals. Some academic doctors were concerned that this does not always happen and that consequently not all patients in the Netherlands are receiving the best available care. In academic hospitals, it was felt, physicians should look for promising new treatment options, preferably for randomized controlled clinical trials within the country. Being informed about investigational treatment options was considered part of the job of an academic medical specialist.

Also, interviewees stressed that (non-standard) treatment decisions are made together with the patient and cited the ideal of shared decision-making. About half of the interviewees felt that when the end of a standard treatment trajectory comes into view, they must inform patients about all available non-standard options, including investigational drugs. During an interview, one physician explained:

From the very beginning here [in the academic hospital], fifteen years ago, we have been in dialogue with patients, and we have said: “We’ve got something [non-standard] for you, you don’t have to take part, what do you think? What do you think of the risks and benefits?” That is … I find it disconcerting that there is this hype currently going on about this [ideal of shared decision-making]. For to me it is self-evident that this is the way you do things. (neurologist)

According to others, it is up to the physician to decide whether or not expanded access is worth pursuing. Interviewees highlighted that although patients can always refuse a treatment (e.g., chemotherapy) and have the right to a second opinion, they cannot claim access to an investigational drug:

They cannot demand access to an investigational treatment. They have no right to say: you must give me that treatment. That is not how it works. (oncologist)

Interviewees felt that physicians should not transfer the responsibility for clinical decision-making to the patient and they should determine themselves whether or not to inform the patient about remaining options. For example, when the patient might already be too ill or when the physician fears that the unapproved drug cannot be obtained (e.g., because of funding issues), they will not tell the patient about the option. These physicians underlined that they sought to provide only “realistic options” to their patients:

So I find it problematic to say: “I am going to try to get this drug for you.” I would only start to say this if I had the idea that I would succeed in getting the drug. (endocrinologist)

Interviewees said that instead of offering investigational treatments, they considered it their responsibility to discuss the nearing end of life with patients and to help patients to come to terms with the prospect of dying. At the end of life, it was felt, patients had better accept that their lives were going to end, spend time with loved ones, and say goodbye properly. Grasping at straws is not a good way to die.

#### Views on Physicians’ Responsibilities Regarding Expanded Access: Questionnaire Results

Questionnaire respondents were asked to indicate the extent to which they agreed or disagreed with six statements about physicians’ responsibilities regarding expanded access (Fig. 1). Sixty-five questionnaire respondents (61.1 per cent) agreed or strongly agreed with the statement that physicians have a responsibility to refer patients who lack satisfactory standard treatment options to more specialized care (Fig. 1). The majority (78.9 per cent) also agreed or strongly agreed that it is the responsibility of physicians to identify clinical trials in which patients who lack curative or life-prolonging treatment options may possibly be enrolled (Fig. 1).

In our questionnaire, 50 per cent of respondents either agreed or strongly agreed with the statement that physicians should inform their patients about existing options to use investigational drugs (fig. 1). The majority (53.3 per cent) of respondents either agreed or strongly agreed that the decision to use an investigational drug should be made by the patient him- or herself (fig. 1). While 20 per cent of respondents felt that it is the responsibility of treating physicians to look for investigational treatment options on their own initiative, 47.8 per cent of respondents did *not* (Fig. 1). Finally, only 23.3 per cent disagreed with the statement that physicians should not prescribe investigational drugs outside of clinical trials (Fig. 1).

## Discussion

Our study is the first to explore the experiences and moral attitudes of physicians treating patients for whom satisfactory standard treatment options are not (or no longer) available, regarding expanded access to unapproved, investigational drugs. In our sample, the level of awareness of existing programmes for expanded access was variable; some respondents and interviewees had very little knowledge of expanded access. This was not unique to physicians working in rural hospitals; we spoke to interviewees in academic hospitals who were not familiar with expanded access. Our respondents and interviewees had little personal experience with requesting expanded access for their patients; while they do treat patients who have run out of options, they rarely pursue expanded access. Also, our interviewees’ moral attitudes were heterogeneous: whereas some physicians considered it a key component of their job to look for investigational treatment options when the end of a standard treatment trajectory comes into view, others had principled objections against pursuing expanded access, citing concerns related to lack of safety and efficacy data, false hope, and the opportunity costs associated with patients’ continued struggles for finding cures. The majority (almost 60 per cent) of our questionnaire respondents felt that investigational drugs should *not* be prescribed outside of clinical trials, and only 20 per cent felt that treating physicians should actively look for expanded access opportunities. We observed principled objections among physicians working in academic hospitals as well as in rural or teaching hospitals.

Some interviewees choose not to inform patients about opportunities for expanded access to facilitate a “good death,” associating it with acceptance and abstinence from life-prolonging treatment. However, it may be paternalistic to impose such an ideal, especially as patients may have opposing preferences. Although sometimes, “appropriate palliative care may be a more suitable option for some patients than access to treatments that may well prove to be ineffective and/or unsafe” (Lewis et al. 2014, 844), patients may feel that they should have a say in such decisions (Bunnik and Aarts 2019). While many of our interviewees support shared decision-making, some doctors do believe that it is their responsibility to decide whether or not to bring up expanded access. Physicians’ decisions whether or not to bring it up may be influenced by patients’ physical conditions but also by doctors’ estimations of patients’ personalities (not all patients are “fighters”). This may be done with an appeal to patients’ best interests, but it may conflict with the medical-ethical principle of respect for patients’ autonomy. Moreover, half of our questionnaire respondents felt that treating physicians should inform their patients about existing options for accessing investigational drugs, even though many of them may not support expanded access in principle and may not look into it—or bring it up—in practice. Thus, there may be a tension between physicians’ acknowledged responsibility to discuss all treatment options, including non-standard treatment options, with their patients and their (limited) willingness to enquire into or cooperate in expanded access.

The classic criteria for expanded access (serious/life-threatening illness; no approved alternatives; not eligible for clinical trials; potential benefits should outweigh the harms) were broadly upheld. Interviewees often spoke of being “with their backs against the wall” and having “no other options” as a reason to consider expanded access. Questionnaire respondents however selected “to improve quality of life,” “gain in health,” “possible prolongation of life,” “confidence in effectiveness,” “pharmaceutical company offers compassionate use programme,” and “request from the patient,” more frequently than “with my back against the wall” as reasons to pursue access to investigational drugs outside of clinical trials. We do not know how to explain this; possibly, the acceptability of “being with one’s back against the wall” as a basis for clinical decision-making varies with the respondents’ backgrounds and professional-cultural environments. The interviewees who reported being with their backs against the wall as a reason to pursue expanded access, were mostly oncologists working in academic hospitals. Questionnaire respondents had more varied backgrounds and worked in more varied hospital types. Respondents selected mostly positively formulated reasons focused on beneficence: prolongation of life or improvement of health or quality of life.

While interviewees supported the classic criteria for expanded access, some did mention that when standard of care is inadequate and associated with poor prognosis, they would wish to use investigational options directly, thereby diverging from a classic criterion. These were physicians who had experience with and positive attitudes towards expanded access. Their expressed wish, at times, to pursue expanded access in lieu of sub-optimal, harmful, or unsatisfactory standard of care stands in remarkable contrast to the theme mentioned by some other interviewees, who were not willing to cooperate with a request for expanded access at all, and diverge from clinical guidelines.

Interviewees had differing opinions of the weight to be attributed to potential harms and burdens. This disagreement is reflected in the literature, where some observers argue that when patients are terminally ill, they may justifiably assign less importance to the (unknown) safety risks of investigational drugs (Walker et al. 2014), whereas other authors stress that dying patients should be equally protected against the potential direct harms associated with the use of unproven treatments (Dresser 2015; Raus 2016; Yang et al. 2015).

Some interviewees claimed that, in principle, they would not depart from evidence-based medicine and thus disapproved of expanded access. When pressed, they explained that they only prescribe drugs that are sufficiently studied, approved for marketing, and recommended in clinical guidelines. When asked, for instance, whether they would use an unapproved drug after the successful completion of a phase III clinical trial, some said they would not, and that they would wait for marketing authorization and/or adaptation of clinical guidelines. It seems that for clinical decision-making, these physicians do not use evidence—as presented in the scientific literature—per se, but rather regulatory or professional statements regarding that evidence. The frequently used “evidence-based medicine” argument may thus have been an *argumentum ad verecundiam* (appeal to authority).

Our interviewees have not pursued expanded access because of lack of knowledge and because of principled objections. Over a quarter of our questionnaire respondents select unfamiliarity with the programmes or “I did not know it was possible” as reasons not to pursue the use of investigational drugs. The subgroup of respondents (n = 43) who did have knowledge of expanded access (because they had either considered it or had first-hand experience with expanded access) reported various practical obstacles, including lack of reimbursement or institutional support, and problems with health inspectorate and pharmaceutical company. These findings mirror practical and financial hurdles expected or observed by experts, as expressed in the literature (Bateman-House 2016; Darrow et al. 2015; Jerome et al. 2016). In addition, we found negative moral attitudes with regard to expanded access among Dutch medical specialists.

When doctors succeeded in expanded access, the hospital-based pharmacist had often been helpful in navigating those hurdles, bringing up the possibility of trying a compassionate use or named-patient programme and facilitating the application process. In our study, it seems that in Dutch academic or teaching hospitals in which a pharmacist incidentally has some experience with expanded access, physicians may be more likely to pursue it for their patients. This raises concerns with regard to equal access to information and opportunities for expanded access. These concerns are exacerbated by potential differences between patients along the lines of socioeconomic status: patients who are more informed and health literate may be more likely to request expanded access and prompt their doctors to pursue such possibilities than patients who are less privileged. In our country, where equal access to healthcare is deemed very important, such disparities may need to be addressed, as demand for expanded access is likely to increase in the future. Policymakers may consider education of physicians about existing opportunities for expanded access, and funding arrangements for approved requests, as possible ways to even out the current expanded access landscape. If reimbursement were in place, physicians might find it easier to pursue, arrange for, and inform their patients about expanded access.

There are other ethical issues that merit further normative scrutiny. There is ongoing discussion, for instance, and lack of evidence, on whether patients benefit from expanded access to unapproved treatments, and consequently, whether or not policy measures should aim at facilitating its uptake. Also, further research might be directed at informed consent for expanded access, the balance of benefits and harms, dealing with uncertainty, and questions related to physicians’ responsibilities (e.g., whether or not patients should be informed about opportunities for expanded access) and payers’ responsibilities (e.g. whether or not expanded access should be reimbursed). Discussion of these issues is beyond the scope of this paper.

### Limitations

There are several limitations to this study. First, the results may not adequately represent Dutch medical specialists’ views and experiences, as the response rate to our questionnaire was extremely low. It is known from Dutch doctors that they do not respond to invitations to questionnaires in large numbers. We did not offer any incentives. Also, IMS Health mistakenly failed to include the Erasmus MC logo in the invitation. Possibly, physicians’ unfamiliarity with the topic further contributed to a low response rate. This may have introduced bias; our results likely reflect the views and experiences of those who are most willing to answer questions about expanded access and thus most interested in the topic. This applies to our sample of interviewees, too. This could mean that the level of knowledge of and experience with expanded access in the general population of medical specialists in the Netherlands is in fact lower than our questionnaire findings suggest. Moreover, the study population of physicians in the interviews and questionnaire were not completely in line with each other. The population of interviewees were physicians who were often more experienced with expanded access, worked in academic hospitals, and treated patients with more complex medical conditions.

Also, further research has yet to elucidate whether the results of this study translate to healthcare systems in other countries. In the United States, for instance, requests for named-patient access must be evaluated by an institutional review board (IRB), which is believed to constitute another major practical hurdle (Borysowski et al. 2017; Fountzilas et al. 2018), while, in the Right-to-Try context, doctors may theoretically go ahead without FDA-approval. The healthcare system in the Netherlands is characterized not only by its universal health coverage but also by its strong moral commitment to solidarity and equal access to healthcare. Also, the country is geographically small and professionals are well-organized and -connected, which may explain the importance that physicians attribute to the clinical guidelines in certain fields (e.g., oncology). Such strong ties may render physicians less likely to deviate from standard of care and pursue expanded access. Moreover, some of our respondents knew of and/or had used the named-patient programme (only) in cases when a particular drug was temporarily unavailable (e.g., due to production problems) and an alternative had to be imported from another country. In the Netherlands, the same regulatory route is used for the import of drugs that are approved in other countries but not submitted for approval in the Netherlands, and for the named-patient use of investigational drugs. In the interviews, however, we focused exclusively on use of the named-patient programme to prescribe *investigational* drugs that are not approved for marketing anywhere in the world. However, such particularities of the Dutch system may have affected some of our results. Thus, our ability to draw global conclusions from this report on a sample of Dutch physicians’ views and experiences, is limited.

## Conclusion

The level of knowledge and experience with regard to expanded access to investigational drugs is highly variable among our respondents: some doctors are not aware of expanded access at all or have no experience with compassionate use or named-patient programmes. Dutch physicians encounter practical barriers, including lack of reimbursement, unwillingness or inability of the pharmaceutical company to supply the drugs, and the time and effort it takes to complete applications for expanded access. The expertise of hospital-based pharmacists may serve as a facilitator to expanded access. Doctors may have negative attitudes and principled objections against expanded access, citing safety risks and false hope, and may choose not to bring up the possibility of expanded access to protect their patients. It is important for patients, patient representatives, and policymakers to know that treating physicians’ moral attitudes or practical constraints may deter them from informing patients with unmet medical needs about existing opportunities for expanded access to investigational drugs.

## Appendix

**Table:** COREQ checklist. The Role of Physicians in Expanded Access to Investigational Drugs: A Mixed-Methods. Study of Physicians’ Views and Experiences in The Netherlands

Domain 1: Research team and reflexivity		
1	Interviewer/facilitator	EB and NA conducted the interviews together
2	Credentials	PhD
3	Occupation	Postdoctoral researchers
4	Gender	Female
5	Experience	EB and NA received postacademic training in Qualitative Research Methods at Erasmus University. EB had prior experience with interview and focus group studies.
6	Relationship established	Interviewees were contacted by NA by email. There were no prior relationships with interviewees.
7	Participant knowledge of the interviewer	Participants knew we worked at the Department of Medical Ethics, Philosophy and History of Medicine at Erasmus MC and were interested in expanded access from an ethical point of view. Participants knew that myTomorrows was involved in the Responsible Innovation project as a private partner.
8	Interviewer characteristics	Interviewers emphasized a neutral stance with regard to expanded access and intellectual independence from the private partner.
Domain 2: Study design		
9	Methodological orientation and theory	Grounded theory
10	Sampling	Purposive sampling, snowball sampling (aimed at diversity among respondents)
11	Method of approach	Email
12	Sample size	14
13	Non-participation	One medical specialist refused participation. When we asked why, we were invited to discuss this specialist’s views informally. He refused participation because he opposed expanded access in principle. We did not include this meeting in our study.
14	Setting of data collection	We met most respondents at their work places. We met one respondent in a home setting.
15	Presence of non-participants	No one else was present
16	Description of sample	6 out of 10 were female; median age was 50.0 years
17	Interview guide	We used an interview guide. The first two interviews served as pilot interviews. The interview guide was adapted after the first two interviews.
18	Repeat interviews	No repeat interviews were carried out
19	Audio/visual recording	Interviews were audiorecorded
20	Field notes	Field notes were made during the interview to remind the interviewers to return to topics later on in the interview
21	Duration	Interviews lasted 45-90 minutes
22	Data saturation	Data saturation was discussed and reached at 14 interviews
23	Transcripts returned	Interviews were transcribed verbatim. Transcripts were not returned for comment and/or correction. Interviewees did receive an email to thank them fort heir participation. They were informed that they could contact the researchers in case they had anything else to say.
Domain 3: Analysis and findings		
24	Number of data coders	The first 3 transcripts were coded independently by 2 coders (EB and NA). Other transcripts were coded by NA. The 12th and 13th interviews were again coded by 2 coders (EB and NA).
25	Description of the coding tree	We do not provide a description of a coding tree as a supplement. Codes were categorised into themes. Themes are discussed in the Results section.
26	Derivation of themes	Themes were derived from the data
27	Software	NVivo version 11 was used for coding and analysis
28	Participating checking	Participants did not provide feedback on the findings
29	Quotations presented	Quotations are presented to illustrate the themes. Quotations are not identified, but the medical specialisation is provided for each quotation.
30	Data and findings consistent	Yes
31	Clarity of major themes	Major themes are presented in separate sections of the Results section
32	Clarity of minor themes	Minor themes are presented within sections on separate major themes
